# Modulating the electrocatalytic activity of *N*-doped carbon frameworks via coupling with dual metals for Zn–air batteries

**DOI:** 10.1186/s40580-022-00308-8

**Published:** 2022-04-12

**Authors:** Jung Hyun Park, Jae-Hoon Shin, Jong-Min Ju, Jun-Hyeong Lee, Chanhee Choi, Yoonhee So, Hyunji Lee, Jong-Ho Kim

**Affiliations:** 1 Department of Materials Science and Chemical Engineering, Hanyang University, Ansan, 15588 Republic of Korea; 2 Department of Chemical and Biomolecular Engineering, University of Illinois at Urbana Champaign, 600 South Mathews Avenue, Urbana, IL 61801 USA

**Keywords:** Dual metal incorporation, *N*-doped carbon framework, Oxygen electrocatalyst, Oxygen reduction reaction, Oxygen-mediated solvothermal radical reaction, Zn–air battery

## Abstract

*N*-Doped carbon electrocatalysts are a promising alternative to precious metal catalysts to promote oxygen reduction reaction (ORR). However, it remains a challenge to design the desired active sites on carbon skeletons in a controllable manner for ORR. Herein, we developed a facile approach based on oxygen-mediated solvothermal radical reaction (OSRR) for preparation of *N*-doped carbon electrocatalysts with a pre-designed active site and modulated catalytic activity for ORR. In the OSRR, 2-methylimidazole reacted with Co and Mn salts to form an active site precursor (MnCo-MIm) in *N*-methyl-2-pyrrolidone (NMP) at room temperature. Then, the reaction temperature increased to 140 °C under an oxygen atmosphere to generate NMP radicals, followed by their polymerization with the pre-formed MnCo-MIm to produce Mn-coupled Co nanoparticle-embedded *N*-doped carbon framework (MnCo-NCF). The MnCo-NCF showed uniform dispersion of nitrogen atoms and Mn-doped Co nanoparticles on the carbon skeleton with micropores and mesopores. The MnCo-NCF exhibited higher electrocatalytic activity for ORR than did a Co nanoparticle only-incorporated carbon framework due to the improved charge transfer from the Mn-doped Co nanoparticles to the carbon skeleton. In addition, the Zn–air battery assembled with MnCo-NCF had superior performance and durability to the battery using commercial Pt/C. This facile approach can be extended for designing carbon electrocatalysts with desired active sites to promote specific reactions.

**Graphical Abstract:**

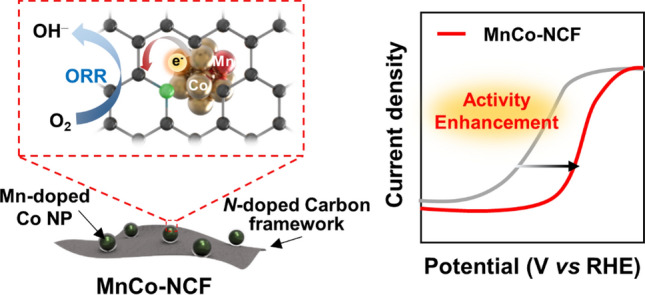

**Supplementary Information:**

The online version contains supplementary material available at 10.1186/s40580-022-00308-8.

## Introduction

Due to increasing energy consumption and environmental concerns, the demand for clean and sustainable energy technologies is growing, and electrocatalytic energy conversion and storage systems have gained much attention in the fields of electric vehicles and wearable devices [1, 2]. Zinc-air batteries are among the more promising energy storage and conversion technologies due to their superior safety, high volumetric energy density, environmental friendliness, and low cost. In Zn–air batteries, oxygen reduction reaction (ORR) is the kernel process occurring on the cathode during discharge. However, ORR suffers from high activation energy and slow kinetics [3–7], requiring effective electrocatalysts to promote it. Platinum electrocatalysts have been widely employed to accelerate ORR. However, the natural scarcity, extremely high cost, and poor stability of platinum have limited the wide practical application of platinum-based electrocatalysts for wearable electronics and electric vehicles. Therefore, it is of great significance to design noble metal-free electrocatalysts effectively promoting the ORR in rechargeable Zn–air batteries.

Carbon-based electrocatalysts are considered promising alternatives to platinum electrocatalysts to promote ORR on the cathode of Zn–air batteries. In particular, heteroatom-doped carbon electrocatalysts were found to exhibit enhanced electrocatalytic activity for ORR through modification of their local electronic structure [8–11]. Furthermore, coupling transition metals onto *N*-doped carbon skeletons was suggested as an effective way to design the active sites of carbon electrocatalysts for ORR [12, 13]. Atomic or nanoparticulate cobalt was frequently incorporated into *N*-doped carbon electrocatalysts to improve their electrocatalytic activity and durability for ORR through further modulation of their electronic structures [14–17]. Previous experimental results and computational simulations revealed that the carbons next to the nitrogen atoms in Co-incorporated *N*-doped carbon electrocatalysts were the active sites for ORR [12, 13]. The effective charge transfer from the Co nanoparticles to the carbon skeleton led to an increase in the electron density of the carbon active sites, resulting in the enhanced electrocatalytic activity of the Co-incorporated *N*-doped carbon electrocatalysts for ORR. In addition, it was reported that doping less electronegative Mn atoms into Co nanoparticles increased the charge density of the resulting Co hybrids [18], eventually facilitating the charge transfer to the *N*-doped carbon sites for the improved electrocatalytic activity in ORR [19]. Although many efforts have been made to understand the active sites of carbon-based electrocatalysts and the fundamental mechanisms for their enhanced activity for ORR, it remains a challenge to design the desired active sites in carbon frameworks in a simple and controllable manner. Hence, it is of great interest to develop a facile approach for synthesis of carbon active sites of which electrocatalytic activity can be effectively modulated by dual metal incorporation for ORR.

Herein, a Mn-coupled Co nanoparticle-embedded *N*-doped carbon framework (MnCo-NCF) was prepared by the oxygen-mediated solvothermal radical reaction (OSRR) of 2-methylimidazole with cobalt nitrate and manganese nitrate in *N*-methyl-2-pyrrolidone (NMP) as an electrocatalyst to promote ORR in rechargeable Zn–air batteries. In our previous study, the OSRR of NMP produced NMP radicals under an oxygen atmosphere at elevated temperature, which then polymerized to produce carbon nanostructures [20]. 2-Methylimidazole formed a complex with cobalt nitrate and manganese nitrate as an active site precursor (MnCo-MIm) in NMP at room temperature [21], which was then involved in the polymerization of NMP radicals generated at elevated temperature (140 °C) under an oxygen atmosphere to produce MnCo-NCF. After complete characterization of MnCo-NCF, its electrocatalytic activity and stability for ORR were investigated and compared with those of a commercial Pt/C catalyst. In addition, the Zn–air battery was assembled using MnCo-NCF, and its performance was examined and compared with that of a battery using Pt/C.

## Experimental methods

### Synthesis of MnCo-NCF

A 17.7 g (0.21 mol) portion of 2-methylimidazole (MIm), 0.9 g (3 mmol) of Co(NO_3_)_2_·6H_2_O, and 77 mg (0.3 mmol) of Mn(NO_3_)_2_·4H_2_O were dissolved in 40 mL of NMP in a 100 mL three-necked round bottom flask with a condenser. The resulting solution was stirred for 1 h at room temperature to form a MIm-metal complex (MnCo-MIm) as an active site precursor. Then, the reaction temperature increased to 140 °C under an oxygen atmosphere for oxygen-mediated solvothermal radical reaction (OSRR) for 24 h. The product was centrifuged at 10,000 rpm for 1 h to obtain the supernatant. A 300 mL portion of ethyl acetate was added into the obtained supernatant (50 mL) to precipitate the desired product, which was collected by centrifuge at 7000 rpm for 15 min. The product was washed with water several times and lyophilized for 12 h. The powder was ground and annealed at 800 °C for 30 min under an Ar atmosphere to obtain the Mn-coupled Co nanoparticles-embedded *N*-doped carbon framework (MnCo-NCF).

Co nanoparticle only-incorporated *N*-doped carbon framework (Co-NCF) was prepared by the same procedure but in the absence of Mn(NO_3_)_2_·4H_2_O in the OSRR.

### General procedure for electrochemical measurement of ORR

All the electrochemical measurements were performed using a potentiostat equipped with a typical three-electrode system, including a glassy carbon rotating disk electrode (RDE, 5 mm diameter) as a working electrode, a Pt wire counter electrode, and an Hg/HgO (20 wt% KOH) reference electrode in an O_2_-saturated 0.1 M KOH solution at room temperature. The potentials of the reference electrode were calibrated based on H_2_ evolution and oxidation reactions, which were referenced to a reversible hydrogen electrode (RHE) using the following well-known equation:

$${\text{E}}_{{{\text{RHE}}}} \,{ = }\,{\text{E}}_{{\text{Hg/HgO}}} \, + \,0.21\, + \,0.059\, \times \,{\text{pH}}$$

where ${\text{E}}_{{{\text{RHE}}}}$ is converted potential vs. RHE, and ${\text{E}}_{{\text{Hg/HgO}}}$ is the potential of a ${\text{Hg/HgO}}$ reference electrode. A catalyst ink was prepared by dispersing 2 mg of each catalyst and 0.2 mg of ketjen black in a mixture solution containing 15 µL of Nafion solution and 485 µL of ethanol through sonication for 10 min. For comparison, Pt/C ink was prepared by dispersing 2 mg of the catalyst in the mixture solution containing 15 µL of Nafion solution and 485 µL of ethanol through sonication for 10 min. Then, a 30 µL portion of the catalyst ink was dropped onto a glassy carbon electrode (RDE) to a mass load of 0.61 mg cm^−2^. For ORR and OER measurements, linear sweep voltammetry (LSV) was conducted at a scan rate of 5 mV s^−1^ using an RDE at a rotating speed of 1600 rpm in an O_2_-saturated 0.1 M KOH solution.

### Assembly and measurement of rechargeable Zn–air battery

Aqueous Zn–air batteries were tested using a home-built battery test cell. A catalyst ink was prepared by dispersing 2 mg of each catalyst and 0.2 mg of ketjen black in 485 µL of ethanol containing 15 µL of Nafion (5 wt%) solution and 2 µL of PTFE (60 wt%) through sonication for 10 min. Then, 480 μL of the catalyst ink was dropped onto a carbon paper with a diameter of 2 cm. The carbon paper was completely dried at 60 °C for 30 min before it was used as an air cathode. The final mass loading of catalyst was 0.61 mg cm^−2^. For comparison, the Pt/C ink was prepared in the same way and then dropped onto a carbon paper.

A Zn plate and a solution mixture of 6 M KOH and 0.2 M Zn(OAc)_2_ were used as anode and electrolyte, respectively, in rechargeable Zn–Air batteries. The fabricated Zn–air batteries were assessed using air as an oxygen source provided by an air pump connected to the air cathode. The charge–discharge profile was recorded using a multichannel battery test system. The period of each charge–discharge cycle was 10 min, with a 2 s rest time per cycle.

## Results and discussion

### Synthesis of MnCo-NCF and identification of its structure

**Scheme 1.**
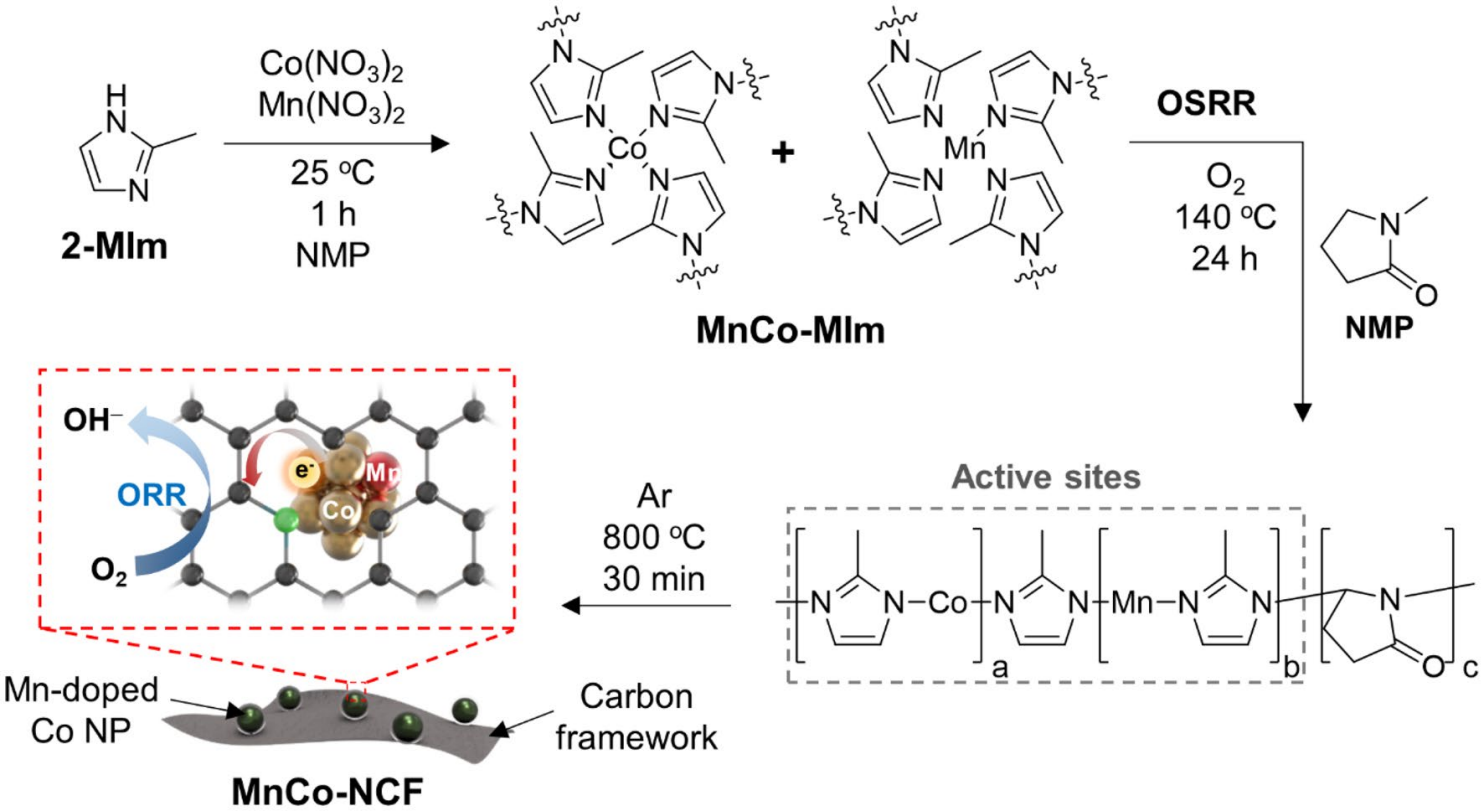
Schematic illustration of the synthesis of a Mn-coupled Co nanoparticle-embedded *N*-doped carbon framework (MnCo-NCF)

To prepare MnCo-NCF, 2-methylimidazole (2-MIm) reacted with Mn(NO_3_)_2_ and Co(NO_3_)_2_ in NMP at room temperature to form a complex (MnCo-MIm) as an active site precursor (Scheme 1). Then, the reaction temperature increased to 140 °C under an oxygen environment to generate NMP radicals, which then polymerized with MnCo-MIm to produce a polymeric form of *N*-doped carbon framework. After annealing under an Ar atmosphere, MnCo-NCF was obtained.

The chemical structure of MnCo-MIm was characterized by Fourier transform infrared (FT-IR) spectroscopy. As shown in Additional file 1: Fig S1, several peak shifts were observed in the FT-IR spectrum of MnCo-MIm. The C=N stretching vibration of an imidazole ring in MnCo-MIm shifted from 1594 to 1576 cm^−1^ compared with that of pristine 2-MIm. Another stretching vibration mode of an imidazole ring shifted from 1436 to 1416 cm^−1^ in the spectrum of MnCo-MIm after forming a MnCo-MIm complex. The shifts of in-plane ring bending (from 1113 to 1141 cm^−1^) and out-of-plane ring bending (741 to 755 cm^−1^) vibrations appeared in the spectrum of MnCo-MIm [22]. The formation of MnCo-MIm was additionally confirmed by X-ray diffraction (XRD). The XRD pattern of MnCo-MIm indicates a ZIF-67-like structure, showing its characteristic peaks for (011), (002), (112), (022), (013), (222), (114), and (044) at 7.29, 10.35, 12.68, 14.65, 16.41, 18.00, 22.08, and 29.57° (Additional file 1: Fig S2), respectively [23]. These spectral features clearly reveal that MnCo-MIm successfully formed as an active site precursor in NMP before the OSRR.

**Fig. 1:**
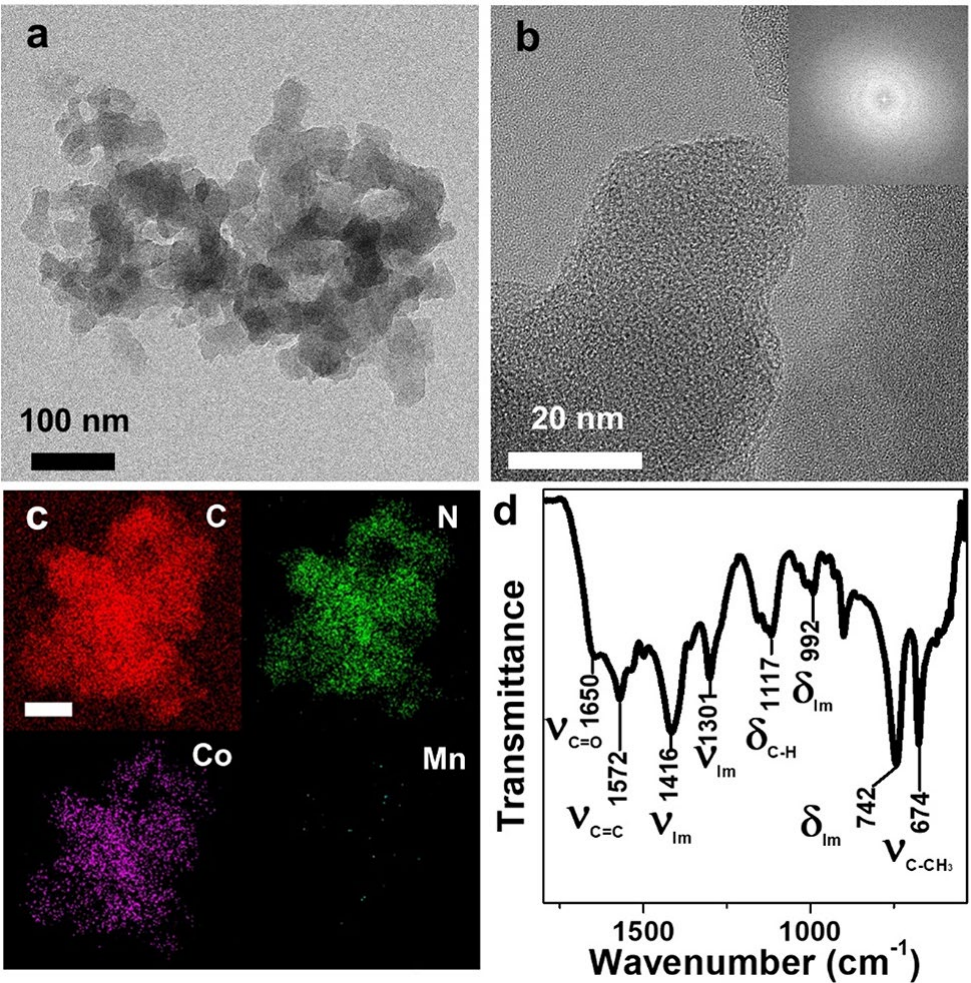
Structure of MnCoNCF before annealing. **a** TEM, **b** HR-TEM (inset: FFT-pattern), and **c** Elemental mapping images of MnCo-NCF precursors (scale bar is 100 nm). **d** FT-IR spectrum of MnCo-NCF before annealing

The MnCo-MIm active site precursor was then involved in the polymerization of NMP radicals produced at elevated temperature (140 °C) under an oxygen atmosphere to form a polymeric form of *N*-doped carbon framework (denoted “MnCo-NCF before annealing”) as shown in Fig. 1a. The as-prepared *N*-doped carbon framework was amorphous according to its transmission electron microscopy (TEM) and fast Fourier transform (FFT) images (Fig. 1b). In addition, atomic cobalt and manganese were uniformly dispersed on the whole skeleton of MnCo-NCF before annealing (Fig. 1c). The amounts of Co and Mn incorporated in MnCo-NCF before annealing were 24.94 and 2.02 wt%, respectively, as measured by inductively coupled plasma-atomic emission spectroscopy (ICP-AES) (Additional file 1: Table S1), indicating that 2-MIm might have a stronger binding affinity to Co than to Mn.

The FT-IR spectrum of MnCo-NCF before annealing clearly shows the characteristic peaks for the stretching vibration and out-of-plane bending modes of an imidazole ring at 1416, 1301, 992, and 742 cm^−1^ (Fig. 1d). The C–CH_3_ stretching of 2-methylimidazole was also observed at 674 cm^−1^ in the spectrum of MnCo-NCF. Moreover, the intense peaks at 1650 and 1572 cm^−1^ for the respective C=O and C=C bonds of NMP in the spectrum of MnCo-NCF suggest that the active site precursor MnCo-MIm was copolymerized with NMP radicals during the OSRR process as depicted in Scheme 1 [24].

The chemical structure of MnCo-NCF before annealing was identified by X-ray photoelectron spectroscopy (XPS) (Additional file 1: Figure S3). The C–C, C–N, C=O, and O–C=O peaks were clearly observed at 284.5, 285.4, 286.3, and 288.6 eV, respectively, in the C1s XPS spectrum of MnCo-NCF. MnCo-NCF showed the characteristic peaks for pyridinic N (398.56 eV), metal-coordinated N (399.90 eV), imidazolic N (400.92 eV), and Co^2+^ (781.07 and 796.75 eV). Furthermore, two weak peaks for Mn^2+^ were observed at 640.75 and 652.24 eV in the Mn2p XPS spectrum of MnCo-NCF before annealing. All these results reveal that the active site precursor MnCo-MIm was successfully incorporated in the *N*-rich carbon skeleton of MnCo-NCF before annealing [13].

As shown in the XRD pattern of MnCo-NCF before annealing, however, the crystalline structure of MnCo-MIm disappeared in the structure obtained after its polymerization with NMP radicals in the OSRR (Additional file 1: Figure S4). We speculate that reactive NMP radicals were sufficiently produced to react with all the MnCo-MIm molecules in the polymerization step of the OSRR, leading to amorphous MnCo-NCF.

**Fig. 2:**
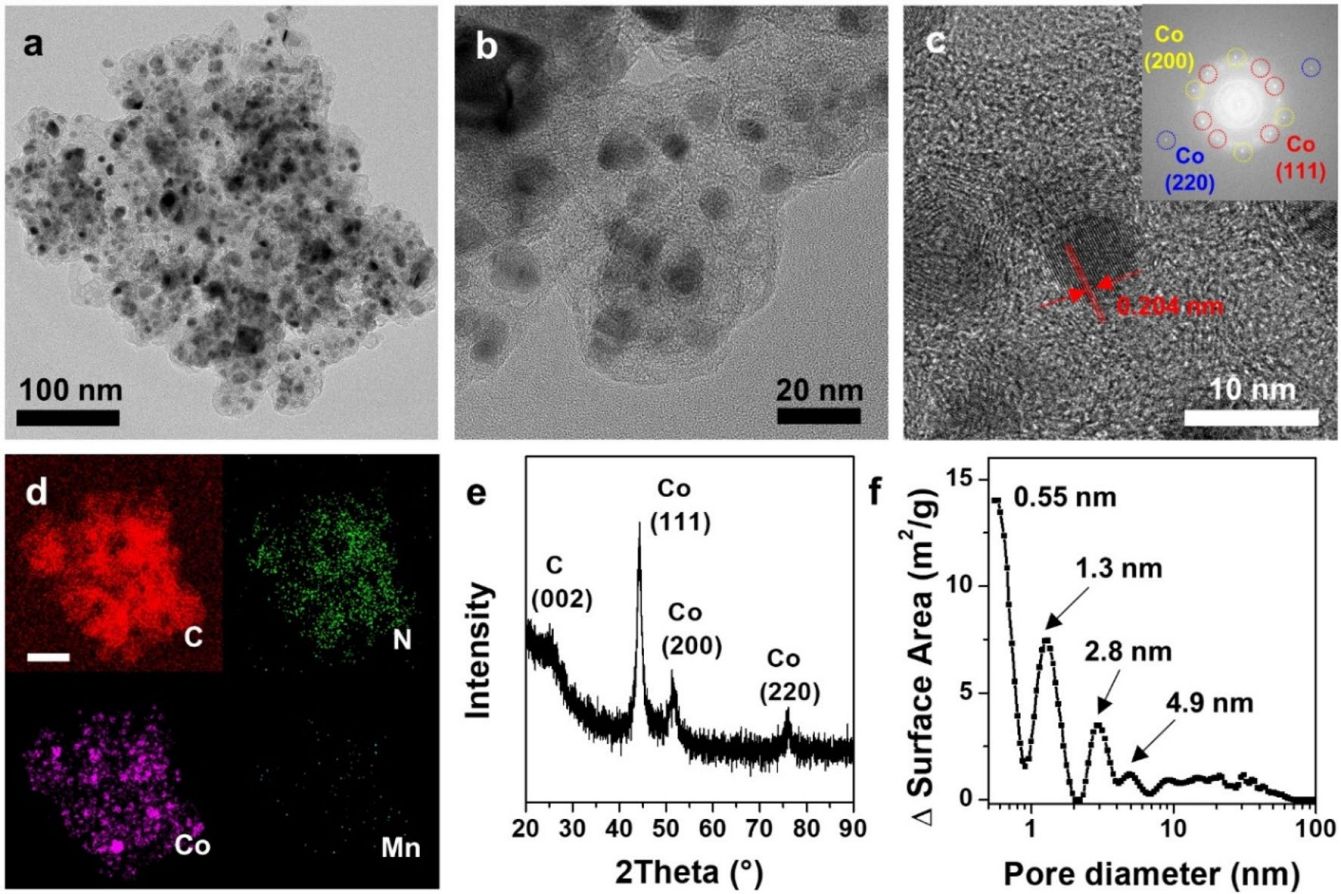
Structural analysis of MnCo-NCF after annealing. **a, b** TEM, **c **HR-TEM (inset: FFT pattern), and **d **Elemental mapping images of MnCo-NCF. **e** Powder XRD pattern and **f** Pore size distribution of MnCo-NCF after annealing

In the thermal treatment of MnCo-NCF, the abundant Co atoms migrated and aggregated to form Co nanoparticles inside the carbon framework (Fig. 2a, b). From the HR-TEM image of MnCo-NCF after annealing in Fig. 2c, the lattice space of 0.204 nm for the (111) facet of Co nanoparticles was clearly observed along with the (200) and (220) facets identified by the FFT pattern. No Mn nanoparticles, however, were formed in MnCo-NCF after annealing. The elemental mapping images of MnCo-NCF show that it retained the uniform dispersion of similar C, N, and Co contents along with a small portion of Mn in the carbon framework after annealing (Fig. 2d).

The powder XRD spectrum of MnCo-NCF clearly confirms the structure of Co and Mn embedded in the carbon framework (Fig. 2e). Diffraction peaks at 44.28, 51.22, and 76.17° correspond to the (111), (200), and (220) facets of Co nanoparticles, respectively, consistent with the FFT pattern of MnCo-NCF. In contrast, there were no characteristic peaks for Mn nanoparticles in the XRD spectrum of MnCo-NCF, indicating that Mn was atomically incorporated into the carbon framework or the Co lattice. MnCo-NCF possessed micropores of 0.55 and 1.3 nm and mesopores of 2.8 and 4.9 nm (Fig. 2f and Additional file 1: Fig. S5). Micropores and mesopores of carbon structures can improve electron and mass transport during electrocatalysis at the solid–liquid interface [25–27]. Hence, the porous structure of MnCo-NCF would be beneficial for increased frequency of collision between molecular oxygen and the active sites, leading to effective oxygen reduction reaction.

### Effect of dual metal incorporation on the electronic structure on mnco-ncf

The effect of dual metal incorporation on the electronic structure of MnCo-NCF was further investigated by XPS. In our previous report, charge transfer from Co nanoparticles to the carbon skeleton was suggested to enhance the electrocatalytic activity of the carbon active site for ORR [12, 13]. To facilitate the charge transfer from the Co nanoparticles to the carbon framework, a less electronegative transition metal Mn was coupled to the Co nanoparticles in MnCo-NCF. For comparison, a Co nanoparticle only-incorporated *N*-doped carbon framework (Co-NCF) was also synthesized through the OSRR in the absence of Mn precursor. Co(NO_3_)_2_ formed a complex with 2-MIm (denoted Co-MIm) in NMP as shown in its XRD pattern (Additional file 1: Figure S2, black line). After Co-MIm underwent the OSRR at 140 °C under an oxygen atmosphere, the amorphous carbon framework (Co-NCF before annealing) was produced as shown in its TEM image and XRD pattern (Additional file 1: Figs S4 and S6). The chemical structure of Co-NCF was confirmed by XPS (Additional file 1: Figure S3e–S3g), and there were no characteristic peaks for Mn in the XPS spectrum of the as-prepared Co-NCF (Additional file 1: Figure S3h). After annealing Co-NCF, Co nanoparticles formed inside the carbon framework (Additional file 1: Figure S6b and S7).

**Fig. 3:**
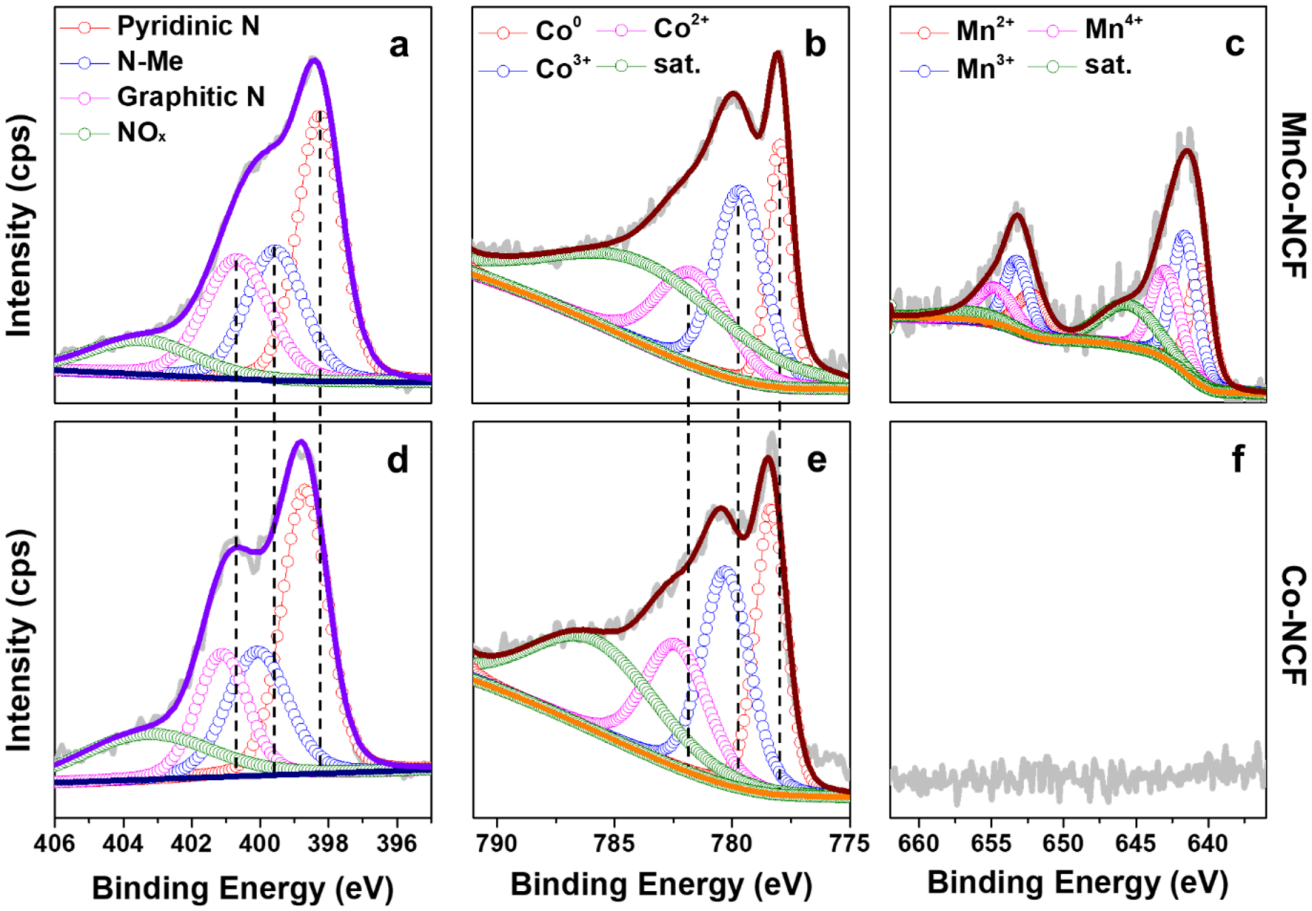
Chemical structure of MnCo-NCF. **a** N1s, **b** Co2p, and **c** Mn2p XPS spectra of MnCo-NCF. **d** N1s, **e** Co2p, and **f** Mn2p XPS spectra of Co-NCF after annealing

As shown in Fig. 3, several shifts in the binding energy of both N1s and Co2p were clearly observed in MnCo-NCF compared with that of Co-NCF after annealing (Fig. 3), which indicates modification of the electronic structure. The Mn2p XPS spectrum shows the Mn oxidation states of 2+, 3+, and 4+(Fig. 3c), reconfirming that a 2.06 wt% portion of Mn was atomically coupled in MnCo-NCF. The atomic incorporation of Mn into MnCo-NCF was observed in FFT and XRD patterns (Fig. 2c and e), showing the absence of Mn nanoparticles. The atomic coupling of Mn induced large shifts in the binding energy of pyridinic *N* (398.7 to 398.3 eV), metal-coordinated *N* (400.1 to 399.6 eV), and graphitic *N* (401.1 to 400.7 eV) in MnCo-NCF compared with those in Co-NCF (Fig. 3a and d). Furthermore, binding energy shifts were obvious in the Co2p XPS spectrum of MnCo-NCF compared to that of Co-NCF. Metallic Co, Co^3+^, and Co^2+^ peaks for Co2p_3/2_ appeared at 777.9, 779.6, and 781.6 eV, respectively, in the XPS spectrum of MnCo-NCF and at 778.4, 780.3, and 782.4 eV in the XPS spectrum of Co-NCF (Fig. 3b and e). The Co peaks for Co2p_1/2_ showed similar binding energy shifts (Additional file 1: Figure S8). The binding energy shift of Co2p shows that Mn was atomically coupled to Co nanoparticles in the MnCo-NCF structure. Coupling a less electronegative Mn into the Co nanoparticles facilitated charge transfer from Co nanoparticles to the *N*-doped carbon skeleton in the MnCo-NCF structure, which induced the binding energy shift in the N1s of MnCo-NCF. The electron-rich carbon framework of MnCo-NCF exhibited enhanced electrocatalytic activity for ORR.

### Electrocatalytic activity of MnCo-NCF for ORR

The electrocatalytic activity of MnCo-NCF for ORR was measured in O_2_-saturated 0.1 M KOH electrolyte and was compared with that of commercial Pt/C and Co-NCF. MnCo-NCF showed a higher limiting current density (5.3 mA/cm^2^) and a larger half-wave potential (0.843 V) for ORR than those of Co-NCF (4.7 mA/cm^2^ and 0.819 V, respectively) (Additional file 1: Fig. 4a). The half-wave potential of MnCo-NCF for ORR was comparable with that of Pt/C (0.861 V). Due to the enhanced charge transfer and hierarchically porous structure of MnCo-NCF, it exhibited a greater increase in electrocatalytic activity for ORR than did Co-NCF.

**Fig. 4:**
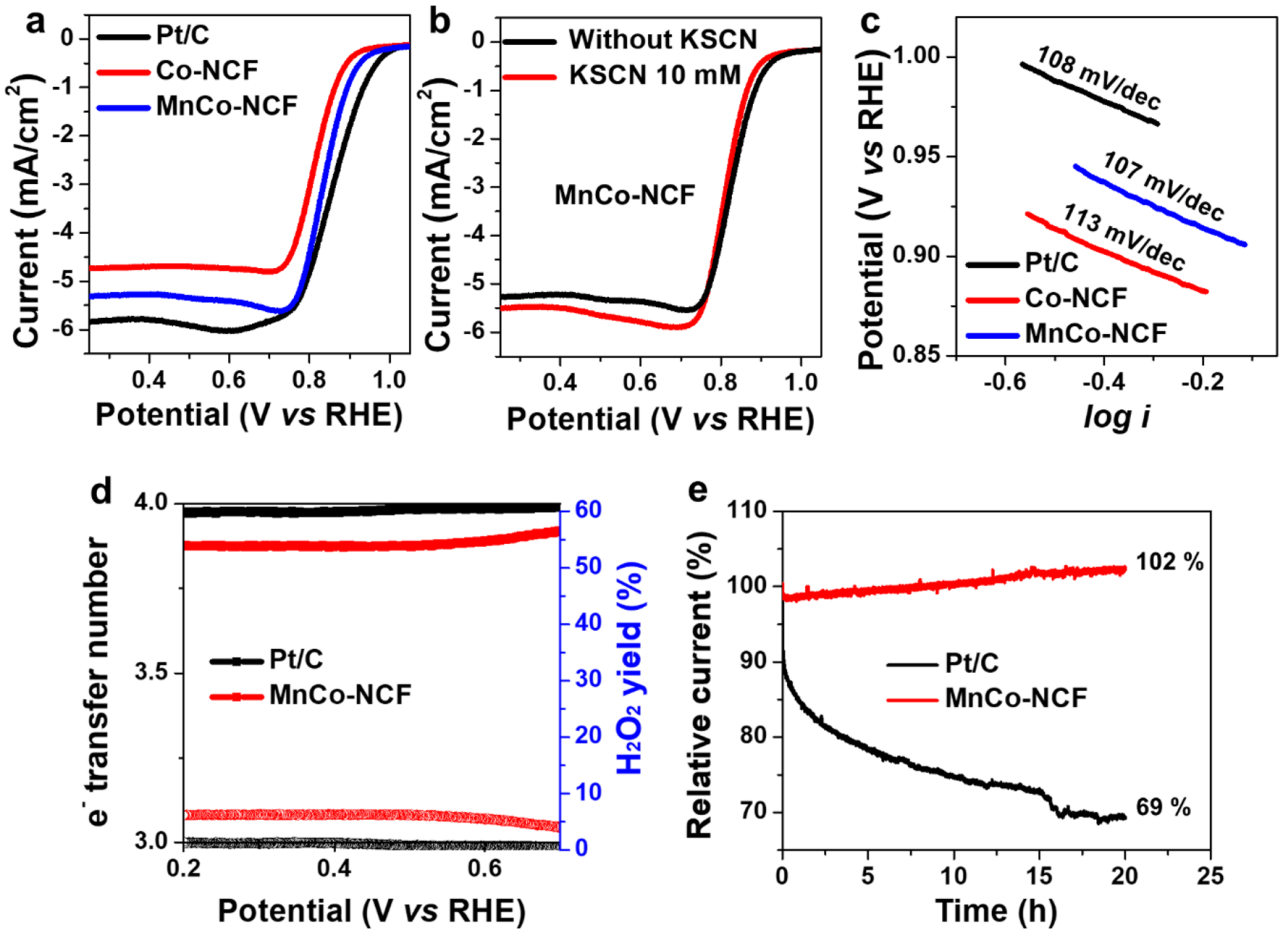
Electrocatalytic activities of MnCo-NCF for ORR. **a** LSV curves of Co-NCF, MnCo-NCF, and Pt/C for ORR in O_2_-saturated 0.1 M KOH solution at 1600 rpm. **b** LSV curves of MnCo-NCF in the presence and absence of KSCN (10 mM), **c** Tafel plots, and **d** Electron transfer numbers and peroxide yields of MnCo-NCF and Pt/C for ORR. **e** Chronoamperometric responses of MnCo-NCF and Pt/C for continuous ORR at 0.6 V (vs RHE)

To identify the ORR active site of MnCo-NCF, whether carbon framework or Co nanoparticle, the electrocatalytic activity was further tested in the presence of 10 mM KSCN that can block metal active sites [28, 29]. As shown in Fig. 4b, however, the electrocatalytic activity of MnCo-NCF for ORR hardly changed even in the presence of KSCN at high concentration, clearly supporting the *N*-doped carbon framework rather than the Co nanoparticle as the active site for the electrocatalytic ORR. The electrocatalytic activity of the carbon active site in MnCo-NCF was enhanced by the improved charge transfer from the Mn-coupled Co nanoparticle to the *N*-doped carbon framework.

To further elucidate the effect of dual metal incorporation on the electrocatalytic activity of MnCo-NCF, the Tafel slopes of the prepared catalysts and Pt/C were compared (Fig. 4c). The Tafel slope of MnCo-NCF (107 mV/dec) for ORR was smaller than that of Co-NCF (113 mV/dec), indicating faster reaction kinetics. The Tafel slope of MnCo-NCF was similar to that of Pt/C (108 mV/dec). The electrochemically active surface area (ECSA) of electrocatalysts is a crucial factor in determining the catalytic activity, which is proportional to the double-layer capacitance of the catalysts. To measure the ECSA of MnCo-NCF, Co-NCF, and Pt/C, cyclic voltammograms in the potential range of 1.16 – 1.26 V (vs*.* RHE) were obtained by varying scan rate, and the difference between anodic and cathodic currents was plotted as a function of scan rate (Additional file 1: Figure S9). The double-layer capacitance of MnCo-CNF was 26.05 mF/cm^2^, while that of Co-CNF was 24.89 mF/cm^2^ (Additional file 1: Figure S10). Both values were much higher than that of Pt/C (9.88 mF/cm^2^). Dual metal incorporation of Mn and Co into the *N*-doped carbon framework did not increase the ECSA. However, it did modulate the electronic structure of the *N*-doped carbon framework to improve the electrocatalytic activity of MnCo-NCF for ORR.

The electron transfer number of MnCo-NCF for ORR was measured using a ring-rotating disk electrode (RRDE) in alkaline media. As shown in Fig. 4d, the electron transfer number was 3.87 with a hydrogen peroxide yield of 6%, suggesting a predominant four-electron pathway in the MnCo-NCF-catalyzed ORR. Most of the oxygen molecules were directly converted into hydroxide without formation of an intermediate H_2_O_2_ in the MnCo-NCF-catalyzed ORR under alkaline conditions. The above values of MnCo-NCF were close to the four-electron transfer values of Pt/C with a peroxide yield of 1.23%.

Excellent stability of electrocatalysts for ORR is required for application in Zn–air batteries. To validate the superior electrocatalytic stability of MnCo-NCF, its chronoamperometric response was measured at 0.6 V (vs RHE) and compared with that of Pt/C (Fig. 4e). MnCo-NCF showed almost constant activity for ORR during a 20-h continuous reaction, while the commercial Pt/C showed a huge decrease in electrocatalytic activity over the same time range. The half wave potential and limiting current density of MnCo-NCF remained almost the same after the chronoamperometry test for 20 h (Additional file 1: Figure S11). This result clearly reveals that MnCo-NCF exhibited outstanding stability in electrocatalytic activity for ORR in alkaline medium.

### Performance of MnCo-NCF-based Zn-air battery

**Fig. 5:**
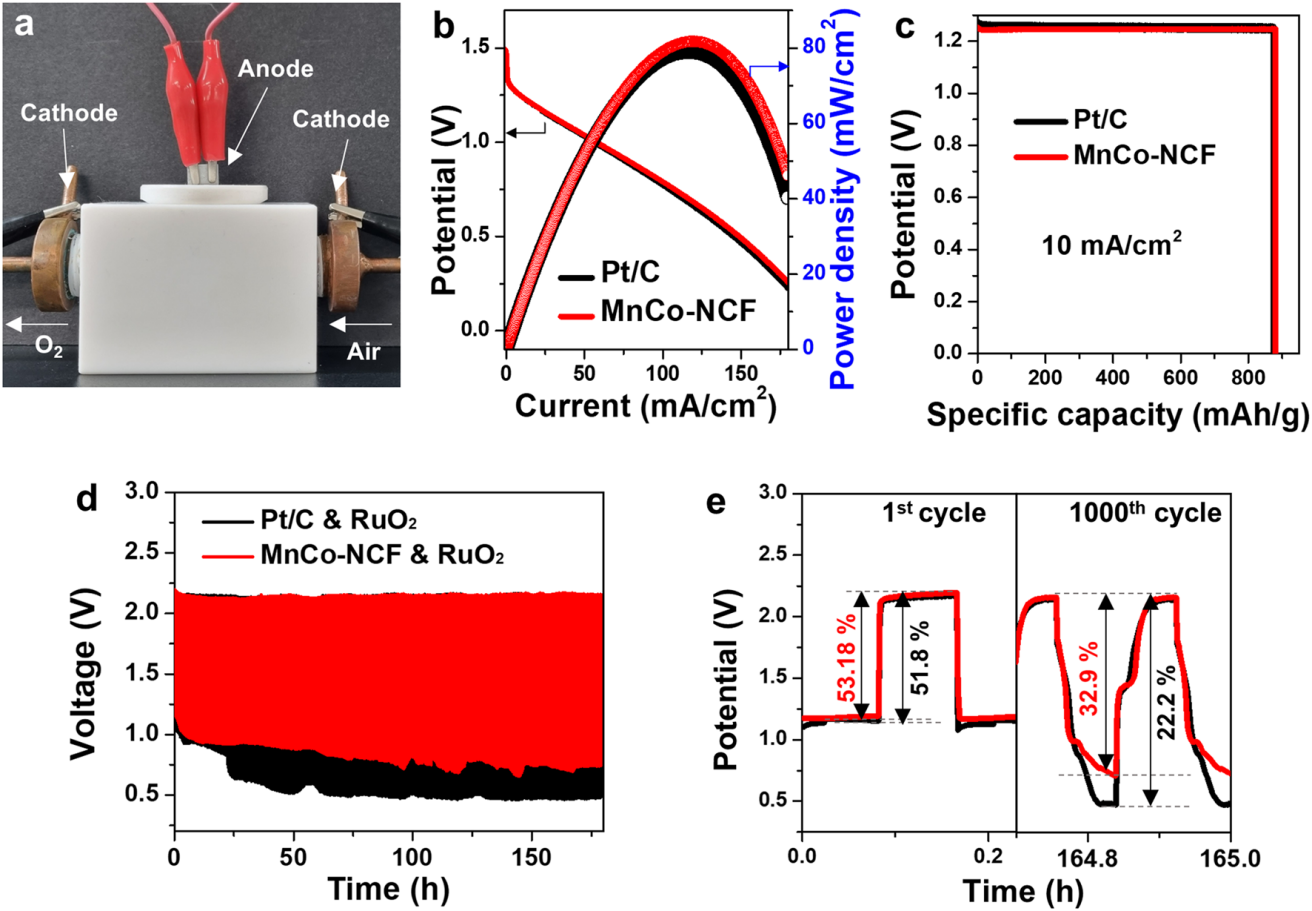
Performance of a rechargeable Zn–air battery assembled with MnCo-NCF. **a** Photograph of the three-electrode Zn–air battery assembled with MnCo-NCF and RuO_2_. **b** Discharge LSV curves and power density plots, **c** Specific capacity, and **d-e** Charge–discharge profiles of the MnCo-NCF-catalyzed Zn–air battery and the Pt/C-catalyzed battery. RuO_2_ was employed as a catalyst for charge in both batteries

MnCo-NCF was employed as a cathode electrocatalyst in an aqueous Zn–air battery composed of three-electrodes: one anode and two cathodes for charge and discharge (Fig. 5a) [30]. One cathode for discharge was prepared by drop-casting MnCo-NCF onto a carbon cloth, and the other for charge was fabricated with RuO_2_ nanoparticles on a carbon cloth. A Zn plate was used as an anode. A 6 M KOH solution with 0.2 M of Zn(OAc)_2_ was employed as an electrolyte, and air was pumped very slowly into the electrode as an oxygen source. As shown in Fig. 5b, the Zn–air battery using MnCo-NCF as a cathode catalyst showed a superior discharge profile with a peak power density of 81.8 mW/cm^2^ compared with the battery using commercial Pt/C (79.2 mW/cm^2^). The specific capacity of the Zn–air battery assembled with MnCo-NCF (880.2 mAh/g) was larger than that of Pt/C (874.2 mAh/g) at 10 mA/cm^2^ with 1.24 V potential (Fig. 5c).

Finally, the durability of Zn–air batteries was assessed through a repeated cycling test with a cycle sequence of 5 min-discharge and 5 min-charge. As shown in Fig. 5d, the Zn–air battery assembled with MnCo-NCF displayed a smaller voltage gap than the battery assembled with Pt/C during the repeated discharge–charge cycle for 170 h. Moreover, the MnCo-NCF-used Zn–air battery had a greater voltaic efficiency (53.18%) at the first discharge–charge cycle than did the Pt/C-used battery (51.8%) (Fig. 5e). The Zn–air battery assembled with MnCo-NCF showed a smaller drop in voltaic efficiency (32.9%) than did the battery with Pt/C (22.2%) after 1,000 cycles. This result reveals that the Zn–air battery using MnCo-NCF as an electrocatalyst for ORR exhibited superior durability to the battery using Pt/C. All the results for electrocatalytic activity and battery performance suggest MnCo-NCF as a promising alternative to expensive noble metal electrocatalysts for ORR.

## Conclusions

MnCo-MIm was readily prepared and employed as an active site precursor in the OSRR of NMP to produce a Mn-coupled Co nanoparticle-embedded *N*-doped carbon framework (MnCo-NCF) for electrocatalytic ORR. MnCo-NCF displayed uniform dispersion of nitrogen and Mn-coupled Co nanoparticles within its whole carbon skeleton. In addition, MnCo-NCF had both micropores and mesopores that can be beneficial for electron and mass transport during ORR. The dual metal incorporation into an *N*-doped carbon framework induced modification of the electronic structure of MnCo-NCF through improved charge transfer from the Co nanoparticle to the carbon skeleton. MnCo-NCF exhibited enhanced electrocatalytic activity for ORR compared with that of the Co nanoparticle only-incorporated *N*-doped carbon framework (Co-NCF), and it had electrocatalytic activity comparable to that of commercial Pt/C. The Zn–air battery assembled with MnCo-NCF showed higher activity and durability than the battery using Pt/C. MnCo-NCF showed its potential as an alternative to noble metal electrocatalysts for ORR. Moreover, this approach for synthesis of a dual metal-incorporated *N*-doped carbon framework with a pre-designed active site can be extended for design of other carbon-based catalysts with desired active sites to promote specific electrocatalytic reactions.

## Supplementary Information

**Additional file 1:** **Figure S1. **FT-IR spectra for 2-methylimidazole (MIm) and MnCo-MIm. **Figure S2. **Powder XRD patterns of MnCo-MIm (red) and Co-MIm (black) before annealing. **Table S1. **The amount of Co and Mn in MnCo-NCF measured by ICP-AES. **Figure S3. a) **C1s, **b) **N1s, **c) **Co2p, **d) **Mn2p XPS spectra for MnCo-NCF before annealing. **e) **C1s, **f) **N1s, **g) **Co2p, **h) **Mn2p XPS spectra for Co-NCF before annealing. **Figure S4. **Powder XRD patterns of MnCo-NCF (red) and Co-NCF (black) before annealing. **Figure S5. **N2 adsorption-desorption isotherms for **a) **MnCo-NCF and **b) **Co-NCF after annealing. **Figure S6. **TEM images for Co-NCF **a) **before annealing and **b) **after annealing. **Figure S7. **Powder XRD pattern of Co-NCF after annealing. **Figure S8. a) **C1s, **b) **Co2p XPS spectra for MnCo-NCF. **c) **C1s, **d) **Co2p XPS spectra of Co-NCF. **Figure S9. **Cyclic voltammograms and plots of a difference (Ja-Jc) between anodic (Ja) and cathodic (Jc) current densities at 1.0 V (vs. RHE) against a scan rate for **a)-b) **Pt/C, **c)-d) **Co-NCF, and **e)-f) **MnCo-NCF. The slope of each profile is twice of double layer capacitance for each catalyst. **Figure S10. **Electrochemically active surface areas (ECSA) of Pt/C, Co-NCF, and MnCo-NCF. **Figure S11. **Polarization curves of MnCo-NCF for ORR before and after 20 h of stability test in 0.1 M KOH solution.

## Data Availability

Not applicable.

## Abbreviations

ORR: Oxygen reduction reaction
OSRR: Oxygen-mediated solvothermal radical reaction
NMP: *N*-Methyl-2-pyrrolidone
MIm: 2-Methylimidazole
Me-MIm: 2-Methylimidazole-metal complex
MnCo-NCF: Mn-coupled co nanoparticles-embedded *N*-doped carbon framework
Co-NCF: Co nanoparticle-only-incorporated *N*-doped carbon framework
FT-IR: Fourier transform infrared spectroscopy
XRD: X-ray diffraction
TEM: Transmission electron microscopy
HR-TEM: High-resolution transmission electron microscopy
FFT: Fast Fourier transform
ICP-AES: Inductively coupled plasma-atomic emission spectroscopy
XPS: X-ray photoelectron spectroscopy
RHE: Reversible hydrogen electrode
RDE: Ring disk electrode
RRDE: Ring-rotating disk electrode
LSV: Linear sweep voltammetry
ECSA: Electrochemically active surface area
