# Supplementary material for: Modulating the electrocatalytic activity of N-doped carbon frameworks via coupling with dual metals for Zn–air batteries
Source: Nano Converg. 2022 Apr 12;9:17. doi: 10.1186/s40580-022-00308-8 (PMC9005593; doi:10.1186/s40580-022-00308-8)
Supplement: Supplementary file 1 — Additional file 1: Figure S1. FT-IR spectra for 2-methylimidazole (MIm) and MnCo-MIm. Figure S2. Powder XRD patterns of MnCo-MIm (red) and Co-MIm (black) before annealing. Table S1. The amount of Co and Mn in MnCo-NCF measured by ICP-AES. Figure S3. a) C1s, b) N1s, c) Co2p, d) Mn2p XPS spectra for MnCo-NCF before annealing. e) C1s, f) N1s, g) Co2p, h) Mn2p XPS spectra for Co-NCF before annealing. Figure S4. Powder XRD patterns of MnCo-NCF (red) and Co-NCF (black) before annealing. Figure S5. N2 adsorption-desorption isotherms for a) MnCo-NCF and b) Co-NCF after annealing. Figure S6. TEM images for Co-NCF a) before annealing and b) after annealing. Figure S7. Powder XRD pattern of Co-NCF after annealing. Figure S8. a) C1s, b) Co2p XPS spectra for MnCo-NCF. c) C1s, d) Co2p XPS spectra of Co-NCF. Figure S9. Cyclic voltammograms and plots of a difference (Ja-Jc) between anodic (Ja) and cathodic (Jc) current densities at 1.0 V (vs. RHE) against a scan rate for a)-b) Pt/C, c)-d) Co-NCF, and e)-f) MnCo-NCF. The slope of each profile is twice of double layer capacitance for each catalyst. Figure S10. Electrochemically active surface areas (ECSA) of Pt/C, Co-NCF, and MnCo-NCF. Figure S11. Polarization curves of MnCo-NCF for ORR before and after 20 h of stability test in 0.1 M KOH solution. [file 40580_2022_308_MOESM1_ESM.pdf]

Additional Information

Modulating the Electrocatalytic Activity of *N*-doped Carbon Frameworks *via* Coupling with Dual Metals for Zn-Air Batteries

Jung Hyun Park[2,†], *Jae-Hoon Shin*[1,†], *Jong-Min Ju*[1], *Jun-Hyeong Lee*[1], *Chanhee Choi*[1], *Yoonhee So*[1], *Hyunji Lee*[1], *and Jong-Ho Kim*[1,*]

[1]Department of Materials Science and Chemical Engineering, Hanyang University, Ansan, 15588, Republic of Korea

[2]Department of Chemical and Biomolecular Engineering, University of Illinois at Urbana Champaign, 600 South Mathews Avenue, Urbana, IL, 61801, USA

[†]*These authors contributed equally to this work.*

* Corresponding author: Jong-Ho Kim, e-mail address: kjh75@hanyang.ac.kr, Tel.: +82-31-400-5275

Contents of Supporting Information:

1. Materials --S2

2. Instruments ---S2

3 Measurement of electron-transfer number and peroxide yield for ORR-----------------------S3

4. Electrochemically active surface area (ECSA) measurement-----------------------------------S3

5. Supporting Figures--S5

Materials

All chemicals were used without further purification. Cobalt nitrate hexahydrate, Manganese nitrate tetrahydrate, 2-methylimidazole, and Nafion solution (5 wt %) were purchased from Sigma Aldrich (USA). *N*-methyl-2-pyrrolidone (NMP), potassium hydroxide, and ethyl acetate were purchased from Daejung Chemicals (Korea), and zinc acetate dihydrate was purchased from Alfa Aesar (USA). Ketjen black, 60 wt % PTFE solutions and potassium thiocyanate (KSCN) were purchased from Mitsubishi Chemical (Japan), Shanghai Aladdin Biochemical Technology (China) and Junsei Chemical (Japan), respectively. Carbon cloth was purchased from Cetech (Taiwan).

Instruments

The morphology and elemental distribution were identified by transmission electron microscope (TEM, JEM-2100F, JEOL, Japan), and crystallinity was collected on a power X-ray diffractometer (XRD, X'Pert-PRO MPD, Malven PANalytical, Netherland) with Cu Kα radiation (λ= 1.5406 Å). Nitrogen adsorption-desorption isotherms were performed at 77 K (Micromeritics, 3Flex, USA). The surface area and pore size distribution were determined from the adsorption and desorption branches by the Brunauer-Emmett-Teller (BET) and Barrett-Joyner-Halenda (BJH) methods. The amount of cobalt and manganese on the catalysts was quantified by inductively coupled plasma-atomic emission spectroscopy (ICP-AES, SPECTRO, SPECTRO ARCOS, Germany). Chemical composition of the catalysts was confirmed by x-ray photoelectron spectroscopy (XPS, AXIS Ultra DLD, KRATOS, UK) and Fourier Transform Infrared spectroscopy (FT-IR, Nicolet iS10, Thermo Scientific, USA). The electrocatalytic activity and charge-discharge performance were tested using a potentiostat/galvanostat (VersaSTAT3, Princeton Applied Research, USA) and a battery cycler (WBCS3000, WonATech, Korea).

Measurement of electron-transfer number and peroxide yield for ORR

The catalyst ink was prepared by dispersing 2 mg of each catalyst in the mixture solution containing 15 μl of Nafion solution and 485 μl of ethanol through sonication for 10 min. Then, a 30 μl portion of the catalyst ink was dropped on a glassy carbon electrode (RDE) to give a mass loading of 0.61 mg cm^{-2}. For comparison, the Pt/C and RuO_2 ink was also prepared using the same procedure. A rotating ring disk electrode (RRDE) measurement was performed for all the catalysts. RRDE consists of a 5.61 mm diameter glassy carbon with a concentric Pt ring at a distance of 318 μm and 7.92 mm diameter, of which current collection efficiency is 0.37. The scan rate of the RRDE was 5 mV s^{-1} and the ring potential was constant at 1.3 V *vs* RHE.

The H_2O_2 yield and electron transfer number (n) during an ORR process were calculated from the following equations;

$$\mathrm{n} = 4\frac{I_d}{I_d + {I_r}/{N}} \qquad \text{(S1)}$$

$$H_2O_2 = 100\,\frac{{2I_r}/{N}}{I_d + {I_r}/{N}} \qquad \text{(S2)}$$

where I_d is a disk current, I_r is a ring current, and N is the current collection efficiency of the Pt ring.

Electrochemically active surface area (ECSA) measurement

The catalyst ink was prepared by dispersing 2 mg of each catalyst and 0.2 mg of ketjen black in the mixture solution containing 15 μl of Nafion solution and 485 μl of ethanol through sonication for 10 min. For comparison, the Pt/C ink was prepared by dispersing 2 mg of the catalyst in the mixture solution containing 15 μl of Nafion solution and 485 μl of ethanol through sonication for 10 min. An electrochemical active surface area (ECSA) reflects the real active surface area of the catalysts for electrocatalytic reactions. The double layer capacitance (C_{dl}) on an electrode-electrolyte interface is proportional to the ECSA. Therefore, C_{dl} was measured by cylic voltammetry (CV). CV measurement with different scan rates from 10 to 25

mVs^{-1} was carried out in the non-Faradaic region from 1.16 to 1.26 V *vs* RHE. The current density variation ($\Delta j = j_a - j_c$) at 1.0 V versus scan rates was plotted. The slope of the plot is twice of C_{dl}. The ECSA of each catalyst can be calculated by the following equation:

$$\text{ECSA} = C_{dl}/C_s \quad \text{(S3)}$$

where C_s is the specific capacitance value of a smooth surface of materials under specific electrochemical condition.

Supporting Figures

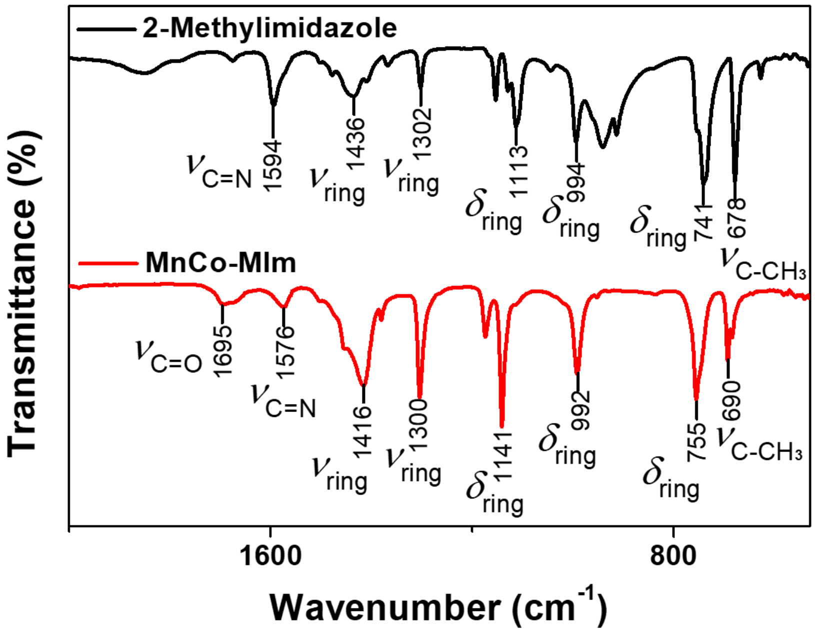

Figure S1. FT-IR spectra for 2-methylimidazole (MIm) and MnCo-MIm.

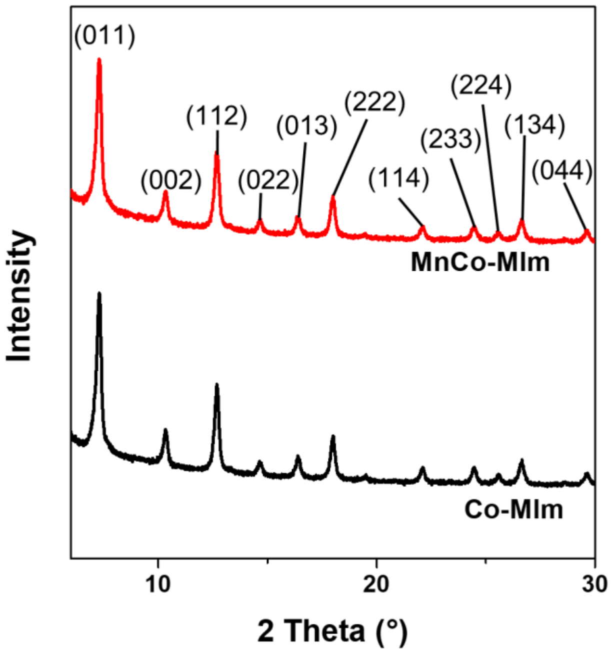

Figure S2. Powder XRD patterns of MnCo-MIm (red) and Co-MIm (black) before annealing.

Table S1. The amount of Co and Mn in MnCo-NCF measured by ICP-AES

	**MnCo-NCF (before annealing)**	**MnCo-NCF (after annealing)**
Co (wt%)	24.94	7.14
Mn (wt%)	2.02	2.06

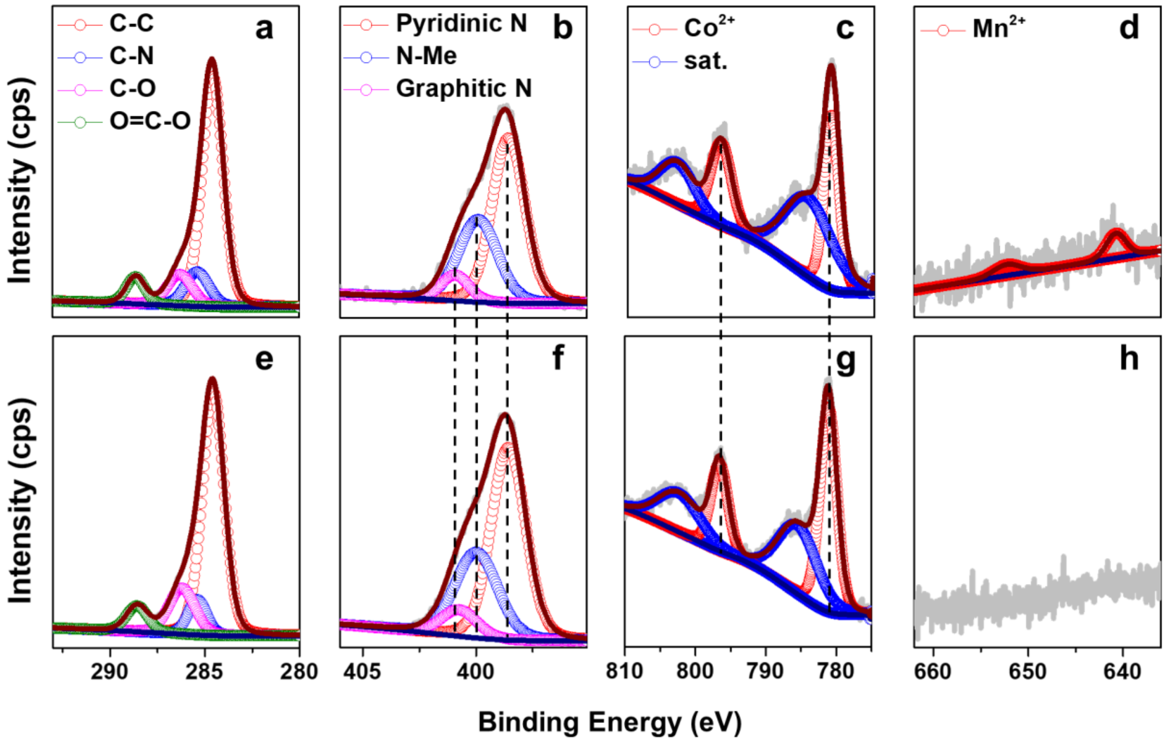

Figure S3. **a)** C1s, **b)** N1s, **c)** Co2p, **d)** Mn2p XPS spectra for MnCo-NCF before annealing. **e)** C1s, **f)** N1s, **g)** Co2p, **h)** Mn2p XPS spectra for Co-NCF before annealing.

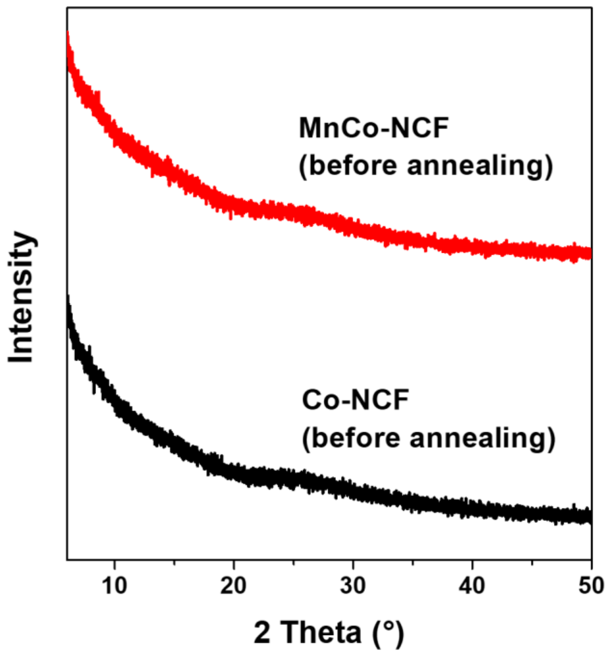

Figure S4. Powder XRD patterns of MnCo-NCF (red) and Co-NCF (black) before annealing..

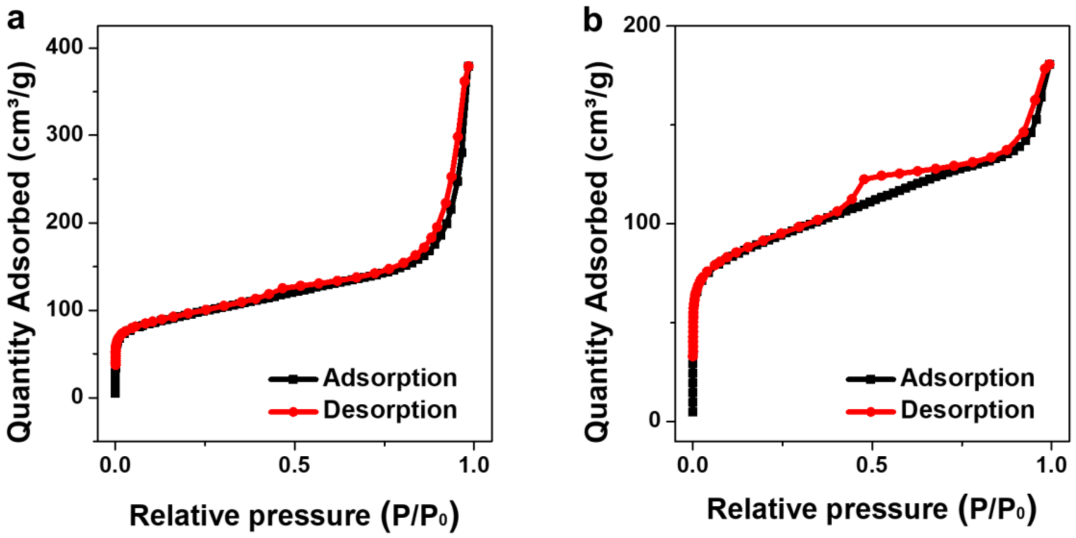

Figure S5. N_2 adsorption-desorption isotherms for **a)** MnCo-NCF and **b)** Co-NCF after annealing.

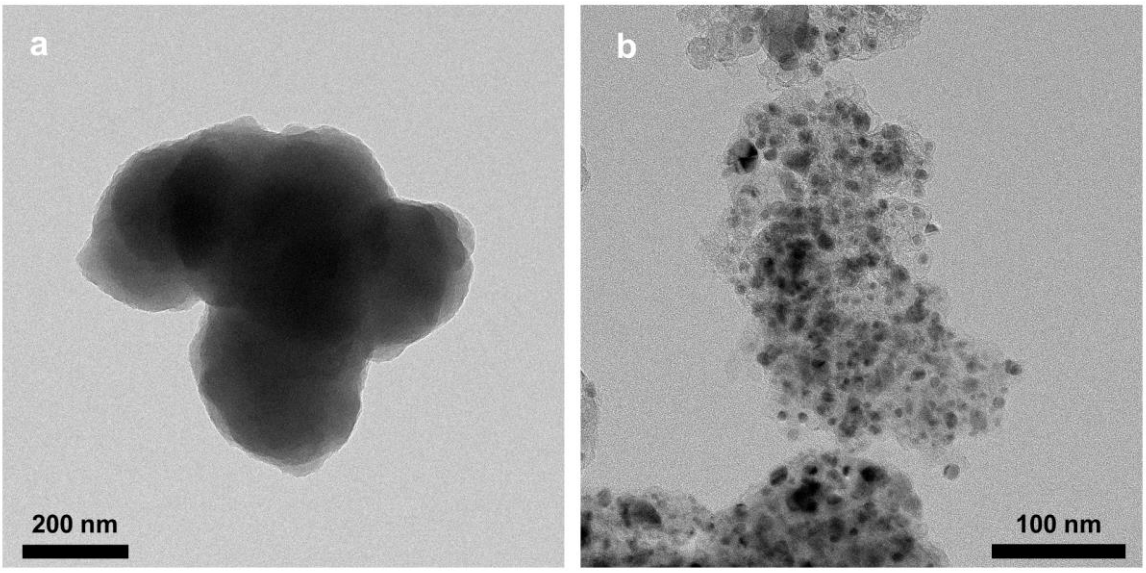

Figure S6. TEM images for Co-NCF **a)** before annealing and **b)** after annealing.

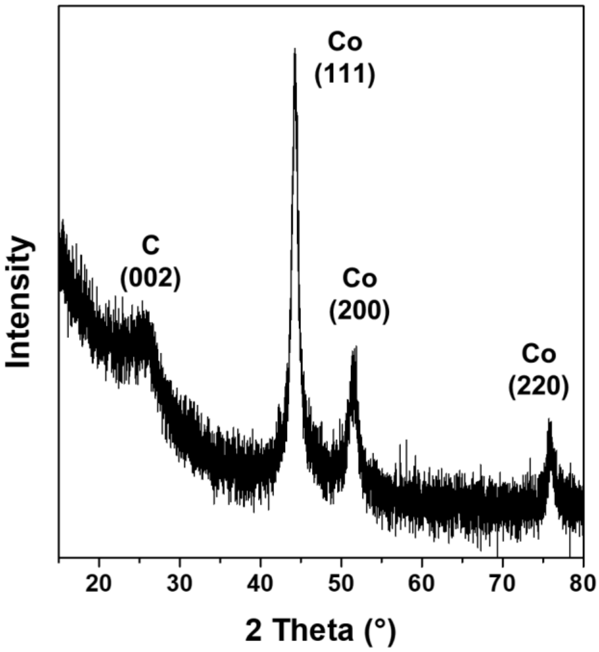

Figure S7. Powder XRD pattern of Co-NCF after annealing.

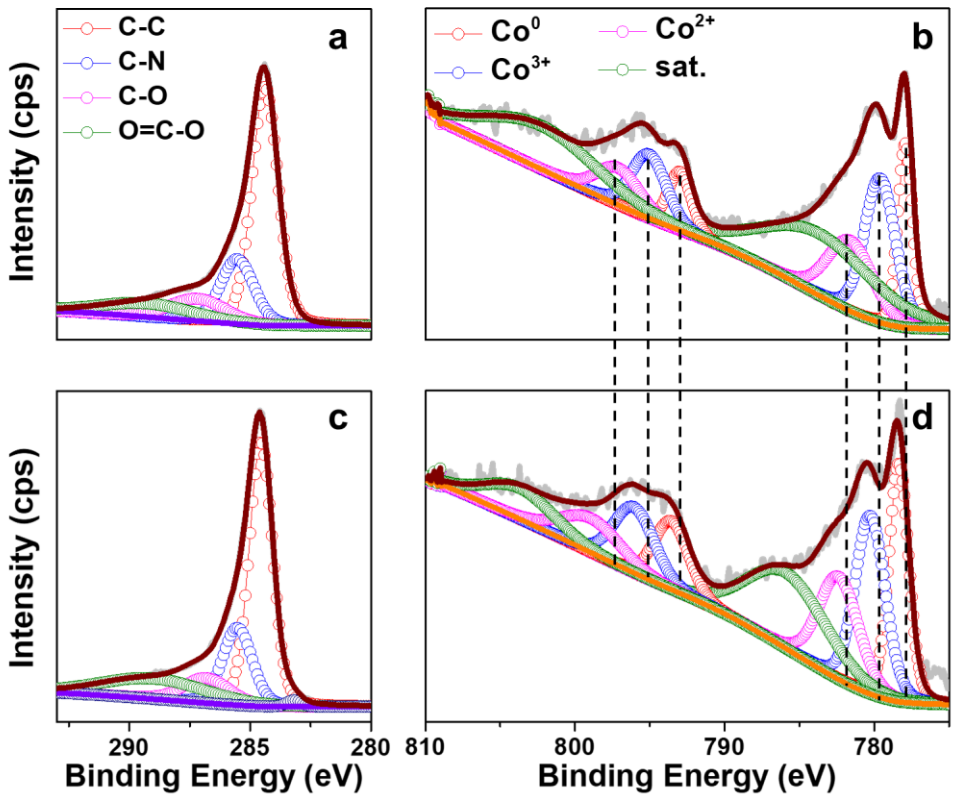

Figure S8. **a)** C1s, **b)** Co2p XPS spectra for MnCo-NCF. **c)** C1s, **d)** Co2p XPS spectra of Co-NCF.

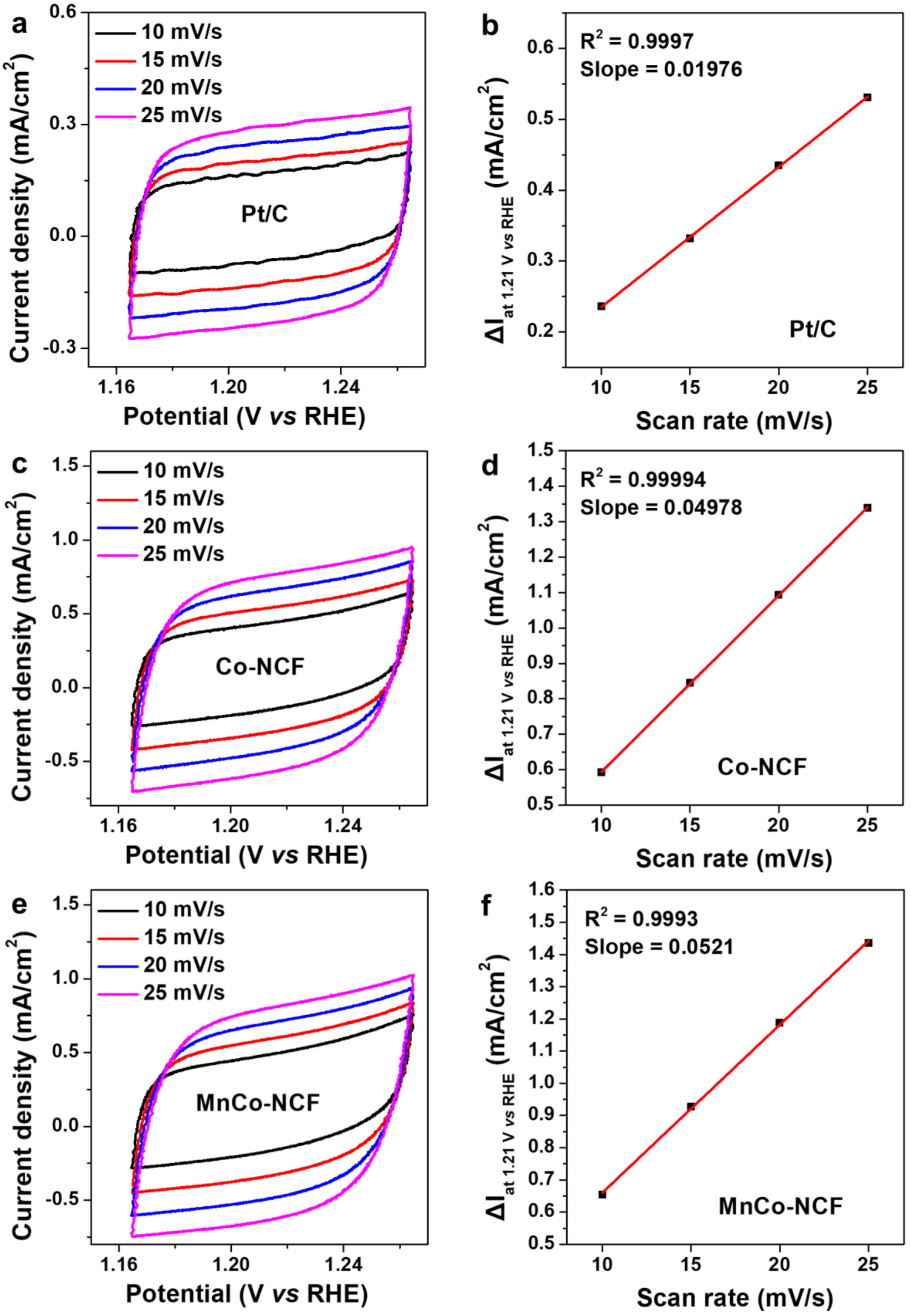

Figure S9. Cyclic voltammograms and plots of a difference (J_a-J_c) between anodic (J_a) and cathodic (J_c) current densities at 1.0 V (vs. RHE) against a scan rate for **a)-b)** Pt/C, **c)-d)** Co-NCF, and **e)-f)** MnCo-NCF. The slope of each profile is twice of double layer capacitance for each catalyst.

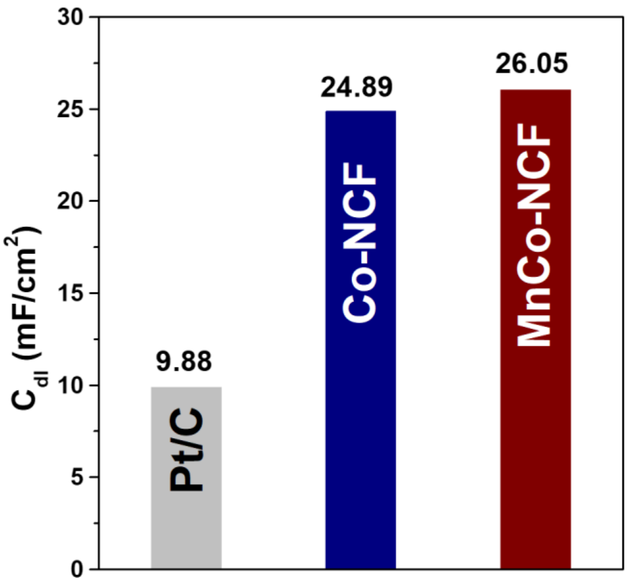

Figure S10. Electrochemically active surface areas (ECSA) of Pt/C, Co-NCF, and MnCo-NCF.

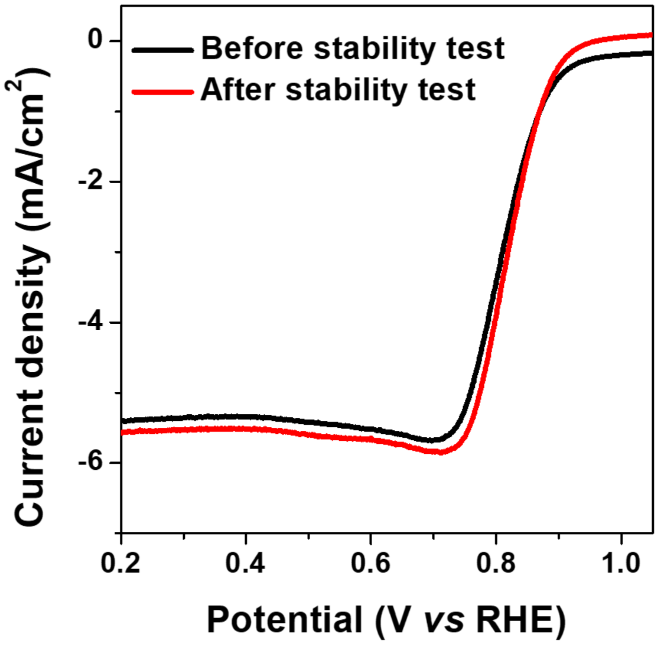

Figure S11. Polarization curves of MnCo-NCF for ORR before and after 20 h of stability test in 0.1 M KOH solution.
